# Analysis of Rheological Properties and Regeneration Mechanism of Recycled Styrene–Butadiene–Styrene Block Copolymer (SBS) Modified Asphalt Binder Using Different Rejuvenators

**DOI:** 10.3390/ma17174258

**Published:** 2024-08-28

**Authors:** Hongmei Ma, Fucheng Guo, Jihong Han, Pengfei Zhi

**Affiliations:** 1 Gansu Provincial Transportation Research Institute Group Co., Ltd., Lanzhou 730030, China; 2 Gansu Province Road Material Engineering Laboratory, Lanzhou 730030, China; 3 Gansu Industry Technology Center of Transportation Construction Materials Research and Application, Lanzhou Jiaotong University, Lanzhou 730070, China

**Keywords:** SBS-modified asphalt binder, rejuvenator, regeneration mechanism, rheological properties

## Abstract

The regeneration performance of an aged styrene–butadiene–styrene block copolymer (SBS) will be significantly influenced by different rejuvenators. The objective of this study was to comparatively investigate the regeneration effect of different SBS-modified asphalt regenerators on aged SBS-modified asphalt. Four types of different regenerant formulations were selected. The optimal rejuvenator content was determined firstly using conventional performance tests. The rheological properties of the aged SBS-modified asphalt binder were evaluated by multiple stress creep recovery (MSCR) experiments. Subsequently, the regeneration mechanism of the SBS-modified asphalt binder was investigated using thin-layer chromatography–flame ionization detection (TLC-FID) and Fourier transform infrared spectroscopy (FTIR). The results showed that the rejuvenator had a certain recovery effect on the penetration, softening point, and ductility of the SBS-modified asphalt binder after aging. The SBS-modified rejuvenating agent was the most favorable among the four types of rejuvenators, where a rejuvenator dosage of 12% showed the optimal rejuvenation effect. The addition of regenerators could appropriately improve the elastic deformation capacity of the aged asphalt binder. The epoxy soybean oil in the regenerant reacted with the aging SBS-modified asphalt binder, supplementing the lost oil in the aged SBS-modified asphalt binder, dispersing the excessive accumulation of asphaltene, and making the residual SBS swell again. The viscoelastic properties of the aging asphalt binder were improved by adjusting the content of components and functional groups to achieve the purpose of regeneration.

## 1. Introduction

Widespread usage is possible for SBS-modified asphalt due to its comprehensive performance in asphalt pavements and its high and low temperature resistance [1,2]. However, SBS-modified asphalt in service will age to varying degrees, resulting in pavement distress. Ultimately, the SBS-modified asphalt pavement will not be able to meet the demands of vehicular traffic due to the rapid growth in traffic and the viscous temperature characteristics of the asphalt material itself [3,4]. Recycling asphalt pavement has emerged as a key field of research in response to the national green low-carbon plan and to encourage the recycling of renewable resources. It will be added to the light-aging SBS-modified asphalt in accordance with Xian et al.’s [5] assessment of the rejuvenating agent in terms of the superior heat resistance, non-pollution, and environmental protection qualities. The study discovered that the rejuvenating agent can control the amount of asphaltene in the aged asphalt and boost its resistance to deformation. The rheological and temperature-sensitive characteristics of furfural extract oil, a black viscous liquid, on aged asphalt were studied by Wu et al. [6]. They discovered that the medium-temperature fatigue resistance and low-temperature cracking resistance of aged asphalt improved with increased furfural extract oil doping from 2% to 10%. The solubilization and dispersion mechanism, the dilution and moderation mechanism, and the wax crystal dispersion mechanism are the three regeneration mechanisms summarized by Yu et al. [7]. The first mechanism means that the polymer surfactants are added to waste asphalt, and they will adsorb onto the surface of asphaltene particles to greatly enhance the stability of the asphalt colloidal system. The second mechanism means that the light waste oil added to waste asphalt can increase the content of solvent components, especially aromatic components, and further has a dilution and blending effect. The last mechanism means that the wax crystal dispersants can disperse wax crystals into finer particles through nucleation and eutectic effects for aged paraffin-based asphalt, which is beneficial for improving the plasticity and deformation resistance of asphalt. Moreover, experimental testing was conducted to demonstrate that different components of the regeneration agents work together in a synergistic way, such as softeners, plasticizers, polymerizing agent, and restorative agents.

Although different rejuvenators were developed to enhance the performance of aged SBS-modified asphalt, the performance and regeneration mechanisms are different. Most of the related research focused on some specific rejuvenators, while the comparative analysis was conducted with a relatively limited effort [8,9,10]. For each specific rejuvenator, the optimized ratio of different components was determined, and the optimal content was recommended based on the basic performance [11], rheological properties [12], adhesion performance [13], etc. Moreover, the regeneration mechanism was also explained using microscopic testing techniques [14,15] and molecular dynamics simulations [16,17]. However, it is difficult to determine which type of regeneration agent has better regeneration performance for aged SBS-modified asphalt in the actual selection of regeneration agents. In conclusion, some studies and research have been conducted on the changes in the physical indices of SBS-modified asphalt after aging, performance degradation, and constituent changes before and after aging [18,19]. However, competitive investigations of different types of rejuvenators of aged SBS-modified asphalt are rarely conducted, and the related regeneration mechanism still needs to be revealed further.

Therefore, the objective of this study is to investigate the effects of aging SBS-modified asphalt performance recovery using various regeneration agents while also combining the characteristics of drought, large temperature differential, and strong ultraviolet light in the Gansu Province of China. Four types of different regenerant formulations are selected. The optimal rejuvenator is determined according to the basic performance. The regeneration effect of the rejuvenators on SBS-modified asphalt and its regeneration mechanism are revealed through the three main testing indicators: multiple stress creep recovery (MSCR) testing, TLC, and FTIR. This provides a foundation for theoretical research on regeneration and the repurposing of SBS + asphalt road surfaces after service.

## 2. Methodology

### 2.1. Rejuvenation Agent

Four types of different regenerant formulations were selected in this study, namely a regenerant based on rubber powder modification, a regenerant based on SBS modification, a regenerant based on styrene–butadiene rubber (SBR) + SBS compound modification, and a regenerant based on SBR + SBS + PPA (polyphosphoric acid) compound modification. PPA was purchased from a company in Lanzhou city, with a concentration of P_2_O_5_ of more than 80%. The experimental schemes are shown in Table 1. The various raw materials were accurately weighed and synthesized according to different schemes. The plasticizer (epoxidized soybean oil), softener (aromatic oil, naphthenic oil), polymerizer, repairer and antioxidant were purchased from a local company and prepared according to the following experimental steps. (1) The weighed epoxidized soybean oil and the softener, plasticizer and polymerization agent were weighed according to their mass ratios and homogeneously mixed in an oil bath at 100 °C to obtain the light component. (2) The repair agent was added to the light component, stirred and continuously sheared for 30 min under the conditions of 160–170 °C and 3000 r/min. (3) Different doses of the antioxidant were added and sheared for 30 min under the conditions of 160–170 °C and 3000 r/min. (4) Different doses of anti-aging components were added and continuously stirred and sheared for 10 min to prepare the regenerating agent, where the temperature remained constant.

### 2.2. Asphalt Binder

Zhenhai 70 # virgin asphalt was selected for the preparation of SBS-modified asphalt, and the performance indicators met the specification requirements, as shown in Table 2.

#### 2.2.1. Preparation of Aged Asphalt Binder

First, in accordance with the requirements of T0610-2011 of the JTG E20-2011 [20], SBS-modified asphalts were subjected to a short-term aging process (RTFOT), where they were heated at 163 °C for 85 min, followed by aging in a pressure-aging vessel (PAV) at 100 °C and 2 bar at 1 MPa for 20 h. By aging in two ways, the aging of the asphalt in production and the asphalt pavement in service conditions was simulated to achieve the effect of heavy aging.

#### 2.2.2. Preparation of Recycled Asphalt Binder

The aged SBS-modified asphalt was placed in an oil bath at 160–170 °C. The self-developed rejuvenators (with the dosage of 4%, 8%, and 12%) were gradually added and artificially mixed for 10 min. Additionally, a digital shear emulsion mixer operating at 3000 rpm for 20 min under shear-mixing conditions allowed for the full integration of the rejuvenator and asphalt aging.

### 2.3. Experimental Test Methods

#### 2.3.1. General Performance Tests

The test temperatures for the needle penetration, softening point, ductility, and kinematic viscosity at 135 °C were chosen to be 25 °C and 5 °C, respectively, in accordance with “T0624-2011” and “T0605-2011” in JTG E20-2011 “Test methods for asphalt and bitumen mixes in highway construction” and “T0606-2011” and “T0619-2011” The essential technical indexes of SBS-modified asphalt in the local standard of Gansu Province’s “Technical specification for highway asphalt pavement construction” (DB62/T3136-2017) are referred to when discussing the performance of SBS-modified asphalt following regeneration.

#### 2.3.2. Multiple Stress Creep Recovery (MSCR) Tests

Asphalt has viscoelastic properties that can be classified as either viscous or elastic. The elastic strain is created when shear creep loading occurs at two different time intervals: instantaneous and delayed. Additionally, asphalt has irreversible strain, which is a component of its viscous properties. The process of repeatedly loading and unloading various loads can be more accurately simulated by the MSCR test [21,22]. The experimental procedure of ASTM D7405-15 (stress control mode, temperature 50 °C, rotor 25 mm) was used to test the viscoelastic characteristics of asphalt. Every asphalt was tested at least once, and after finding three sets of data with small mistakes, the test results were retested for significant inaccuracies. Two creep stress levels, 0.1 kPa and 3.2 kPa, were selected for continuous testing, with 10 cycles at each stress level and a total test time of 200 s.

The viscoelastic properties of asphalt materials can be quantified using the relevant Equations (1) to (5). They can be used to distinguish the elastic properties of asphalt. In accordance with the ASTM D7405-15 calculation formula, the strain recovery rate *R* of the asphalt material and the unrecoverable creep flexibility $J_{\mathrm{nr}}$ were calculated, and the results of the calculation can be used to evaluate the delayed elastic recovery performance of modified asphalt. The formula for determining the external force released after the non-recoverable strain-to-stress ratio is as follows: the greater the recovery rate of asphalt material, the better its ability to resist deformation. *R* denotes the ratio of the recoverable strain of the external force applied and released to the maximum strain during the loading process, usually expressed as a percentage. This phenomenon is somewhat improved by the addition of a recycling agent. The value of the recycled SBS-modified asphalt non-recoverable creep flexibility falls, while the value of the creep recovery rate rises. This suggests that the elastic deformation capacity of aged asphalt can be sufficiently increased by adding a recycling agent.

(1) $$J_{\mathrm{nr}}\left(\sigma ,N\right)=\varepsilon_{u}/\sigma$$

(2) $$R\left(\sigma ,N\right)=100\%\times \left(\varepsilon_{P}-\varepsilon_{U}\right)/\varepsilon_{P}$$

(3) $$J_{\mathrm{nr}}=\sum_{N=1}^{10}J_{\mathrm{nr}}\left(\sigma ,N\right)/10$$

(4) $$R=\sum_{N=1}^{10}R\left(\sigma ,N\right)/10$$

(5) $$J_{nr\text{-}diff}=100\%\times \left(J_{\mathrm{nr}3.2}-J_{\mathrm{nr}0.1}\right)/J_{\mathrm{nr}0.1}$$

where:

σ is the applied stress level, 0.1 kPa and 3.2 kPa, respectively;
$\varepsilon_{u}$ is the unrecovered strain, dimensionless;
N is the loading cycle, times;
$\varepsilon_{P}$ is the peak strain, dimensionless;
$J_{\mathrm{nr}}\left(\sigma ,N\right)$ is the irrecoverable creep softness at the *N*th loading cycle, kPa^−1^;
R(σ,N) is the elastic recovery rate at the *N* loading cycle, %;
$J_{\mathrm{nr}}$ is the average irrecoverable creep softness, kPa^−1^;
*R* is the average elastic recovery rate, %;
$J_{nr\text{-}diff}$ is the stress sensitivity parameter, %.

#### 2.3.3. Microanalysis Test

The changes in the chemical constituents of 70# asphalt and SBS-modified asphalt before and after aging were observed and tested by thin-layer chromatography–flame ionization detection (TLC-FID). Toluene was used to prepare the asphalt solution (5 mL of toluene dissolved 0.1 g of asphalt to configure the asphalt solution), n-heptane was used as the first expansion solution, toluene/n-heptane (80/20 by volume) was used as the second expansion solution, and toluene/ethanol (55/45 by volume) was used as the third expansion solution. The data of the four components were analyzed using the SIC-480 II 5.0 software supplied with the TLC-FID.

FTIR was used to observe and test the changes in the functional groups of SBS-modified asphalt, aged SBS-modified asphalt, and recycled SBS-modified asphalt. FTIR was performed using a Magna-IR750 Fourier transform infrared spectrometer with a scanning range of 4000~400 cm^−1^ wavelengths, several scans of 32, and a resolution of 4 cm^−1^. The temperature of the instrument was controlled at 17~27 °C and it was kept dry, and the IR spectra obtained were processed and analyzed using OMNIC 8.3.103 software.

## 3. Results and Discussion

### 3.1. Regeneration Performance Analysis

Different programs (1#, 2#, 3#, 4#) of rejuvenating agent were added to the aging SBS-modified asphalt. The dosages were 4%, 8%, and 12% of the asphalt mass. The three basic performance indicators of asphalt and kinematic viscosity at 135 °C were used for the evaluation of the effect of asphalt rejuvenation, and the test results are shown in Table 3.

Table 3 shows how the amended asphalt ages, with a noticeable decrease in the penetration and ductility and an increase in the softening point. This suggests that although the modified asphalt’s low temperature resistance decreases with age, its high stability is enhanced. The asphalt treated by SBS presents considerable changes in its three aging indices upon the addition of the regenerant, namely the softening point is decreased and the penetration and ductility are increased.

**Figure 1:**
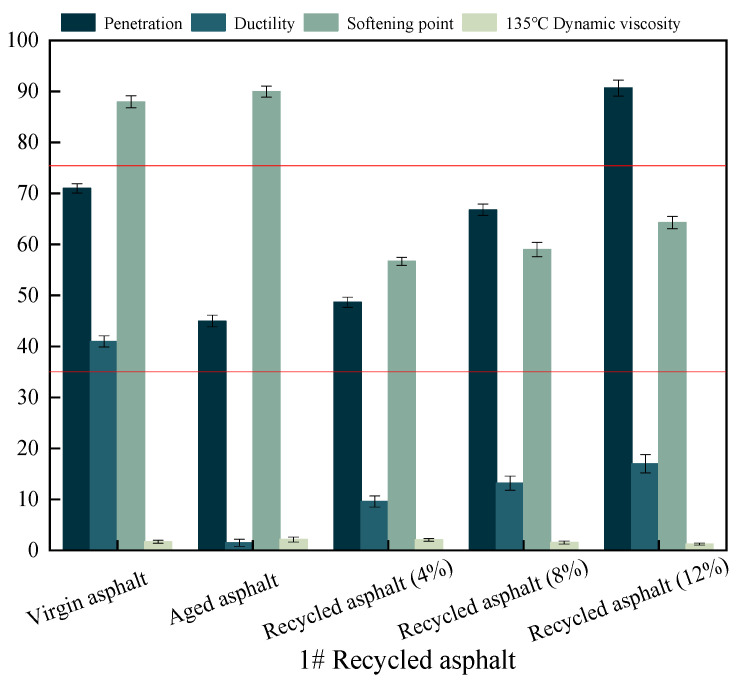
Variations of three basic performance indicators for 1# recycled asphalt, where red line represents the technical requirements.

As shown in Figure 1, the ductility and softening point of 1# recycled asphalt with three different rejuvenating agent dosages do not meet the technical requirements of SBS-recycled asphalt in the local standard “Technical Specification for Construction of Highway Asphalt Pavement” (DB62/T3136-2017) of Gansu Province (5 °C ductility ≥ 35 cm, softening point ≥ 75 °C).

**Figure 2:**
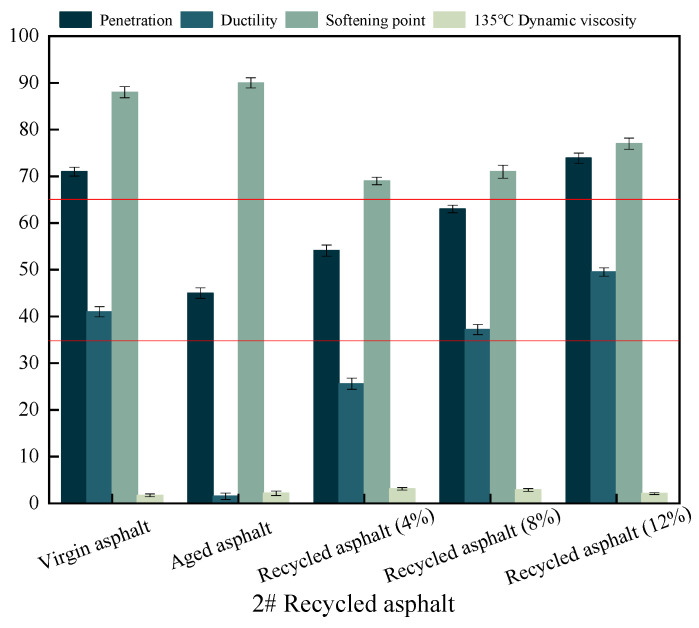
Variations of three basic performance indicators for 2# recycled asphalt, where red line represents the technical requirements.

Figure 2 illustrates how the SBS-modified asphalt gradually reverts to its original form when the amount of recycled material increases. Notably, at 12%, the SBS-modified asphalt exhibits better ductility and penetration than its original form, meeting the standard specifications (≥35 cm for ductility at 5 °C and 60–80 mm for penetration). Although there has been some improvement, the softening point has not returned to its initial level. Overall, the result is satisfactory enough to satisfy the specification criteria.

**Figure 3:**
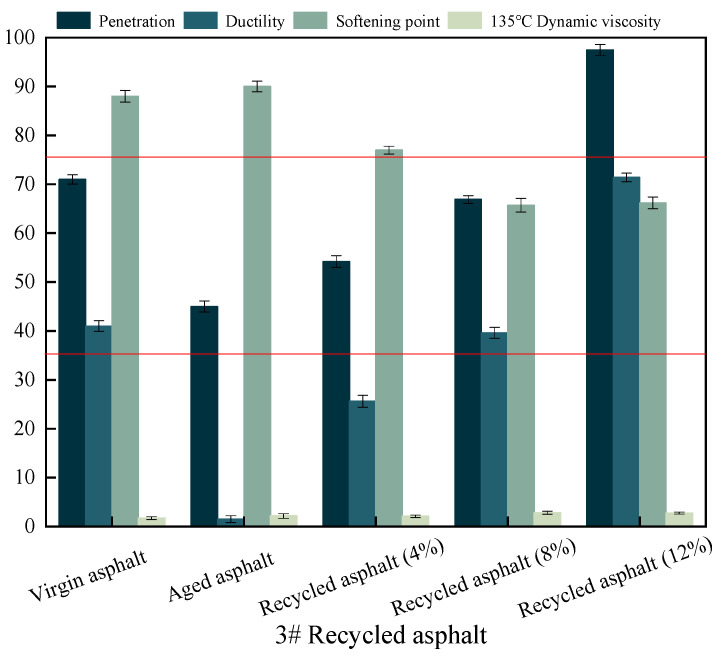
Variations of three basic performance indicators for 3# recycled asphalt, where red line represents the technical requirements.

The recycled asphalt meets the standard requirements for elongation at 5 °C, as seen in Figure 3, with a rejuvenating agent dose of more than 8%. However, the softening point is not restored to its original level and does not meet the standard standards. The viscosity of the SBS-modified asphalt ages and increases. Four types of rejuvenating agents are added once the viscosity of the dosage shows a trend of decreasing, with one type of rejuvenating agent reducing the largest rejuvenating agent dosage of 8% and one type reclaiming the asphalt viscosity close to the original asphalt. The 12% reclaimed asphalt viscosity reduction from the 2# and 3# rejuvenator dosages is higher, but it is still below the initial asphalt level.

**Figure 4:**
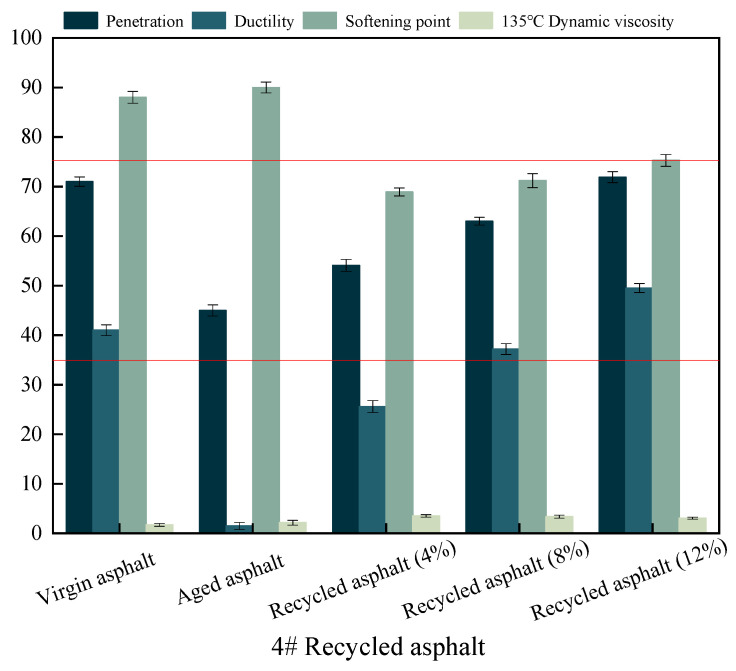
Variations of three basic performance indicators for 4# recycled asphalt, where red line represents the technical requirements.

The viscosity of the reclaimed asphalt does not meet the technical requirements for SBS-reclaimed asphalt in the local standard of Gansu, Technical Specification for Road Asphalt (DB62/T3136-2017) (1.8–3/Pa.s), as illustrated in Figure 4, even though the three primary parameters of the reclaimed asphalt, at 12%, meet the specifications. In summary, 12% of a 2# recycling agent is the ideal mix design.

### 3.2. Analysis of MSCR Trial Results

**Figure 5:**
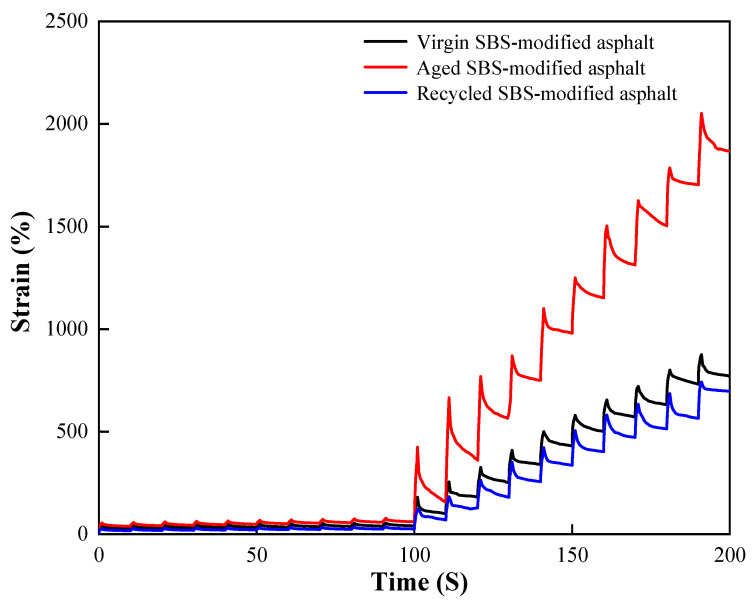
Repeated creep time–strain curve of SBS-modified asphalt.

Figure 5 and Table 4 present the findings of the MSCR test parameters, which were determined using Equations (1)–(5). The optimal outcome was chosen based on the above trial results, and the 2% reclamation agent was applied at 12% of the mass of the aged SMA.

Based on the strain magnitude connections in Figure 5, it is evident that the three asphalts strains exhibited a stepwise increasing tendency over time. The aged SBS-treated asphalt had a much higher strain than the unmodified asphalt. In the meantime, it was nearly impossible to reverse the deformation of the old SBS-modified asphalt. Every test cycle lacked the elastic component. By adding recycling, the old asphalt was less stressed and essentially returned to nearly its original state. It is evident that as the SBS-modified asphalt aged, its elastic qualities declined [23]. However, the effect was somewhat mitigated by the addition of a rejuvenating agent [24]. This is in line with the findings in Table 4 from the previous investigations [25,26].

The J_nr-diff_ can be used to assess how sensitive asphalt materials are to the degree of stress. It can be observed from Table 4 that the SBS-treated asphalt as received had the lowest J_nr-diff_ value, just 14.6% at both stress levels. This implies that the degree of irrecoverable creep flexibility in the original asphalt decreased as the stress increased. To put it another way, the original SBS-modified asphalt creep performance is better suited to circumstances involving significant stress variations [27,28]. The original SBS-modified asphalt’s and the recycled SBS-modified asphalt’s stress sensitivity differences have been enhanced when compared to the aged SBS-modified asphalt’s creep performance.

The greater the value of J_nr_, which indicates the ratio of irretrievable strain to stress following the removal of external forces, the higher the recovery rate of asphalt materials and the higher their ability to withstand deformation. It is evident from the results in Table 4 that, after aging, the SBS-modified asphalt had a much higher J_nr_ value. This suggests that aging strengthens the asphalt modified with SBS’s resistance to deformation and increases its performance at high temperatures. Aged asphalt becomes more resistant to deformation at high temperatures when a recycling agent is added [29]. The J_nr_ value of the SMA grew 2.5 times after aging at 0.1 kPa stress; however, it decreased 54% following the addition of a regenerant.

### 3.3. Analysis of Aging and Regeneration Mechanism of Modified Asphalt

**Figure 6:**
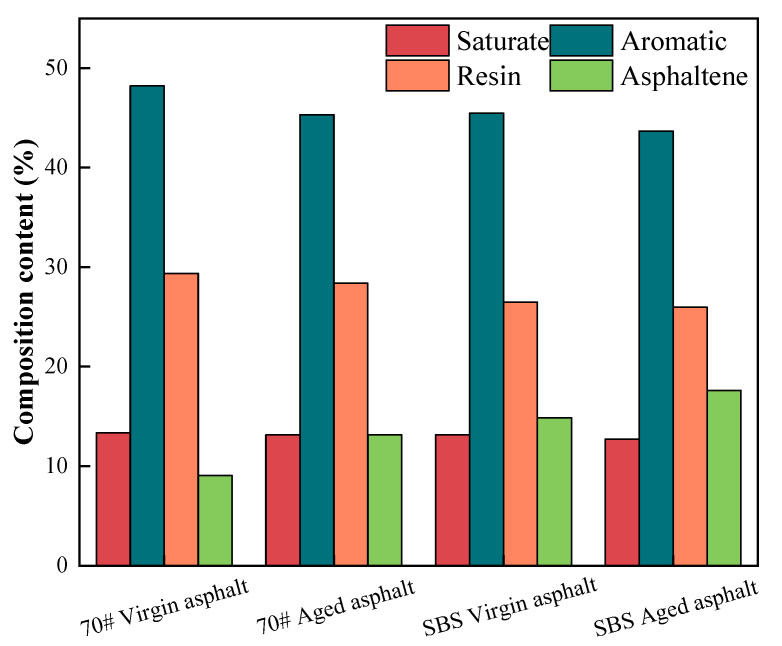
Changes in the four components of asphalt before and after aging.

**Figure 7:**
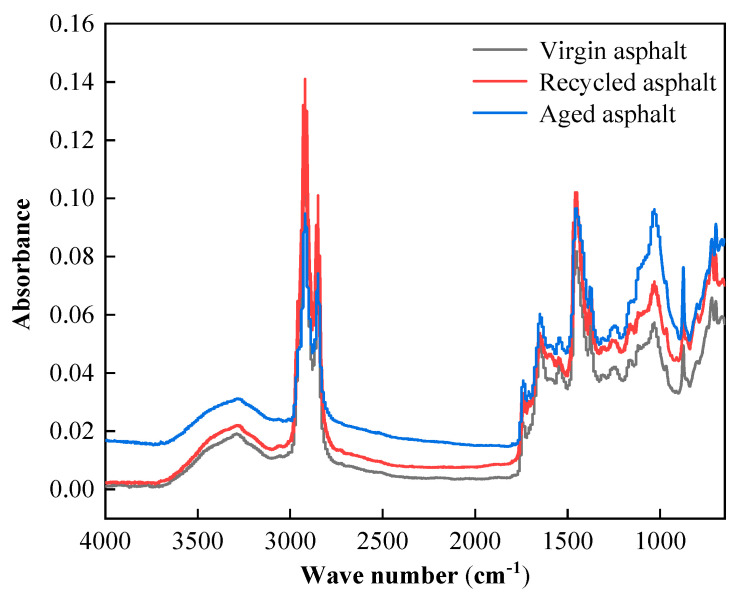
Infrared spectra of SBS-modified asphalt before and after aging and recycling.

To investigate the aging and rejuvenation mechanisms of asphalt, the four components of asphalt and the results of the infrared spectral analysis are shown in Figure 6 and Figure 7.

Figure 6 illustrates the four-component content of SBS-modified asphalt and 70# virgin asphalt prior to aging. The addition of the SBS modifier led to a notable rise in the asphaltene content (from 9.05% to 14.87%) and a minor decrease in the saturated and aromatic fractions. The gums stayed largely unaltered [30]. Because part of the dispersing agent was being absorbed by the modifier, the asphalt’s viscosity increased [31]. Both the SBS-modified asphalt and the 70# base asphalt exhibited an increasing trend in asphaltene content during aging. The rubber mass fraction, aromatic fraction, and saturated fraction all steadily dropped. This resulted from rubber’s higher sensitivity to temperature fluctuations and propensity to react with other components among the four components of asphalt [32]. It has been noted that the temperature influences the conversion of the aromatic fraction to gum, which then turns into asphaltene. Thus, through several conversion pathways, gum and the aromatic fraction eventually turn into asphaltene [33]. During the aging process, the rate of asphaltene growth in the SBS-modified asphalt (18.6%) was less than the base asphalt asphaltene change rate (45.2%). This suggests that the slowing down of the asphalt aging rate relative to the base asphalt was caused by the addition of the SBS modifier.

The microchemical properties of SBS-modified asphalt can be qualitatively analyzed during aging and regeneration by comparing the changes in the peak position and peak value of some characteristic functional groups of asphalt. This will allow for a deeper exploration of the aging and regeneration mechanisms of SBS-modified asphalt from a microscopic perspective. The results of the Fourier infrared spectroscopy presented in Figure 7 show that, in comparison to the initial SBS-modified asphalt, the aging SBS-modified asphalt at 966 cm^−1^, which corresponds to the butadiene C=C bond absorption peaks, appeared to reduce to some extent [34,35]. This suggests that the SBS degradation in the SBS-modified asphalt is occurring during the thermo-oxidative aging process. The distinctive peak in the recycled SBS-modified asphalt vanished, signifying a specific reaction involving the regeneration agent and a portion of the C=C bond, where the absorption peak at about 1600 cm^−1^ corresponds to the benzene ring in the C=C bond. The absence of the absorption peak in the recycled SBS-modified asphalt suggests that the same process may have taken place. The aging SBS-modified asphalt and the plasticizer in the recycling agent are thought to have a relevant response. A distinctive new absorption peak that corresponds to saturated aliphatic aldehydes appeared in the recycled SBS-modified asphalt at 1745 cm^−1^ [36].

The component reconciliation theory, compatibility theory, and rubber plasticization theory are some of the theories of asphalt rejuvenation [37,38]. According to the component-matching theory, the light component will eventually turn into asphaltenes because of aging, increasing the asphaltene content. Therefore, adding an aromatic-rich regeneration agent will help restore the performance of the aging asphalt [39]. Secondly, the compatibility hypothesis states that asphalt is made up of soft asphaltene as a solvent and asphaltene as a solute, which combine to form a polymer solution. During the aging process, the solubility parameter difference between soft asphaltene and asphaltene will grow [40,41]. The road performance of the aged asphalt can therefore be restored by reducing the difference between the two solubility parameters. Third is the rubber plasticization theory, which holds that as asphalt ages, its oil gradually changes into asphaltenes, strengthening the reticulated structure of the molecules and causing embrittlement. To accomplish the goal of asphalt regeneration, the reticulated structure needs to have the right amount of oil added to it to restore the macromolecules’ gel-soluble role. According to the experimental findings, there was a higher dispersion between the tiny molecules because of the degradation of the network structure stability of the SBS-modified asphalt with age. Additionally, there was some degree of decomposition of the SBS molecules and asphalt molecules. Aged SBS-modified asphalt hence performs quite poorly in terms of rejuvenation. The addition of the reclamation agent appeared to influence the binding of broken-down tiny molecules, as evidenced by the infrared spectroscopy results. The rejuvenating agent had a high oil content, which helped to regulate the components of the old asphalt by dispersing the over-aggregated asphaltenes, resolving the residual SBS, and supplanting the aromatic molecules in the aged asphalt. As seen via the increase in ductility, this further enhanced the aged asphalt’s low temperature qualities. Nevertheless, the low softening point of the recovered bitumen was somewhat lowered by the addition of the light component, hence decreasing its high temperature resistance.

## 4. Conclusions

The rejuvenating effect of four different rejuvenator formulations was evaluated and analyzed by conventional performance tests, MSCR tests, TLC-FID, and FTIR. These tests verified the rejuvenating effect of SBS-modified asphalt and revealed its rejuvenating mechanism. The main conclusions were the following:

(1) The addition of a recycling agent to improve the viscosity of aging virgin asphalt and low-temperature resistance asphalt was demonstrated by the significant improvement in the needle penetration and 5 °C ductility of the recycled SBS-modified asphalt with an increasing dose, while the softening point decreased. A thorough comparison of four different modifying agent programs was conducted. The best program was determined by looking at the performance of various properties close to the level of the original asphalt, the three main indicators, the 135 °C kinematic viscosity, and the 2# regenerant dose of 12%.

(2) The aged SBS-modified asphalt lost its elastic properties. The unrecoverable creep flexibility decreased, but the deformation resistance at high temperatures increased. This phenomenon was somewhat improved by the addition of a recycling agent; the value of the recycled SBS-modified asphalt’s non-recoverable creep flexibility *J_nr_* fell, while the value of the creep recovery rate R rose. This suggests that the elastic deformation capacity of aged asphalt can be sufficiently increased by adding a recycling agent.

(3) When examining the aging and regeneration mechanisms of asphalt from a microscopic perspective, the findings indicate that the process of transferring components from asphaltene to colloid is fundamentally what causes asphalt to age. By enhancing the dispersion of the SBS modifier in the aged asphalt and balancing out the light components in the aged asphalt, the regeneration agent helped to accomplish the goal of regenerating the asphalt.

## Figures and Tables

**Table 1:** Composition and dosage of the regenerant in different schemes.

Schemes	Rejuvenator Name	Component	Percentages Included (%)
1#	Rubber powder modification rejuvenator	Softeners	45
		Plasticizers	20
		Polymerizing agents	5
		Aluminate coupling agents	1
		Restorative	2
		Anti-aging ingredients	3
		Rubber powder 40 mesh	5
2#	SBS-modified rejuvenating agent	Softeners	60
		Plasticizers	20
		Polymerizing agents	10
		Aluminate coupling agents	3
		Restorative	10
		Antioxidant BHT	2
		Ultraviolet absorber UV-326	2
		SBS	10
3#	SBR + SBS-modified blend rejuvenating agent	Softeners	60
		Plasticizers	20
		Polymerizing agents	15
		Aluminate coupling agents	3
		Restorative	15
		SBR	10
		SBS	20
4#	SBR + SBS + PPA (polyphosphoric acid) complex modification rejuvenator	Softeners	70
		Plasticizers	20
		Polymerizing agents	10
		Restorative agents	10
		SBR	5
		SBS	25
		PPA	7

**Table 2:** Basic performance indexes of asphalt.

Asphalt	25 °C Penetration (0.1 mm)	Ductility (cm)	Softening Point °C
70# virgin asphalt	74	>100 (15 °C)	48.5
SBS-modified asphalt	71	41 (5 °C)	88

**Table 3:** Test results of different regenerant programs.

Asphalt Binder	Dosage of Regenerant	Penetration at 25 °C (0.1 mm)	5 °C Ductility (cm)	Softening Point (°C)	135 °C Kinematic Viscosity (Pa.s)
SBS-modified asphalt	0%	71	41	88	1.71
Aging of SBS-modified asphalt	0%	45	1.5	90	2.14
1#	4%	48.7	9.6	56.7	2.07
	8%	66.8	13.2	59.0	1.53
	12%	90.7	17.0	64.3	1.25
2#	4%	54.1	25.6	69.0	3.09
	8%	63.0	37.2	71.0	2.87
	12%	73.9	49.5	77.0	2.08
3#	4%	54.2	25.6	65.7	2.82
	8%	66.9	39.6	66.2	2.74
	12%	97.5	71.4	65.6	2.23
4#	4%	54.1	25.6	68.9	3.52
	8%	63.0	37.2	71.2	3.36
	12%	71.9	4905	75.3	3.07

**Table 4:** Modified asphalt MSCR test results.

Asphalt Type	J_nr_/(kPa^−1^)		R/(%)		J_nr-diff_/ (%)
	0.1 kPa	3.2 kPa	0.1 kPa	3.2 kPa	
Virgin SBS-modified asphalt	0.206	0.236	60.3	55.4	14.6
Aged SBS-modified asphalt	0.506	0.586	42.6	36.1	15.8
Recycled SBS-modified asphalt	0.233	0.268	49.5	46.8	15.0

## Data Availability

The original contributions presented in the study are included in the article, further inquiries can be directed to the corresponding author.
